# Characterization of the Gut Microbiome of Patients with *Clostridioides difficile* Infection and Healthy Individuals in Greece

**DOI:** 10.3390/pathogens15030275

**Published:** 2026-03-03

**Authors:** Dimitra Mougiou, Georgia Gioula, Lemonia Skoura, Fani Minti, Theodoros Karampatakis, Dimitrios Malandris, Konstantinos Pelekoudas, Melania Kachrimanidou

**Affiliations:** 1 Department of Microbiology, Medical School, Aristotle University of Thessaloniki, 54124 Thessaloniki, Greece; ggioula@auth.gr (G.G.); fani.minti@yahoo.com (F.M.); melinaka@auth.gr (M.K.); 2 Department of Microbiology, AHEPA University Hospital, 54636 Thessaloniki, Greece; lemskour@auth.gr; 3 Department of Microbiology, Papanikolaou General Hospital of Thessaloniki, 57010 Thessaloniki, Greece; tkarampatakis@yahoo.com; 4 Department of Internal Medicine, Papanikolaou General Hospital of Thessaloniki, 57010 Thessaloniki, Greece; dimitris_malandris@hotmail.com (D.M.); kwstaspele1994@gmail.com (K.P.)

**Keywords:** *Clostridioides difficile*, gut microbiome, CDI, bacterial diversity, microbial abundance

## Abstract

Background/Objectives: The gut microbiome plays an important role in the colonization of an individual by *Clostridioides difficile* and in the development of *Clostridioides difficile* infection (CDI). The main purpose of this study was to compare the gut microbiomes of patients with CDI and healthy individuals. Methods: We prospectively included 48 individuals: 32 patients with CDI and 16 healthy individuals. Microbiomes were analyzed by sequencing the hypervariable regions of the 16S rRNA gene using an Ion GeneStudio™ S5 System. Further statistical analysis of microbiome data was performed with the open-source programming language R version 3.5.2. Results: Among the CDI patients, Firmicutes and Proteobacteria were the most abundant phyla, while *Enterobacteriaceae* and *Enterococcaceae* were the most abundant families. Genus-level analysis showed that *Enterococcus* was the dominant genus in CDI patients; in contrast, in healthy individuals, *Faecalibacterium* was the most abundant. The MaAsLin2 tool revealed that members of the family *Enterococcaceae* and the genus *Enterococcus* were more abundant in patients with CDI than in healthy individuals. Alpha and beta diversity did not reveal differences between the two study groups. Conclusions: We observed differences in microbiome patterns between healthy individuals and CDI patients that were consistent with the literature. Further studies are needed.

## 1. Introduction

*Clostridioides difficile* (*C. difficile*) is a Gram-positive, anaerobic, spore-forming bacillus that colonizes the gastrointestinal tract of humans and other animals [1,2,3]. It has been identified as the primary and most prevalent cause of nosocomial antibiotic-associated diarrhea worldwide. *C. difficile* exists in two forms: a metabolically active, vegetative cell and a dormant, antibiotic-resistant spore. *C. difficile* spores are thought to be the main cause of transmission through the fecal–oral route because they are resilient, common, and capable of surviving in the presence of oxygen. However, a spore’s ability to colonize the gut, germinate into a vegetative cell, and produce one or more toxins—such as toxin A (TcdA), an enterotoxin; toxin B (TcdB), a cytotoxin; and, less frequently, binary toxin (CDT)—is necessary for the development of disease [4,5,6].

CDI is regarded as predominantly hospital-acquired, though there has been a marked increase in community-acquired cases in recent years [5]. CDI presents a wide range of manifestations, varying from asymptomatic colonization and mild self-limiting diarrhea to life-threatening colitis and toxic megacolon [7]. The mortality rate attributed to CDI is 5%, but it can reach 15–20%, especially in intensive care units, owing to complications caused by the infection [8].

*C. difficile* is the causative agent of CDI in humans. The infection is associated with changes in the gut microbiota that are primarily due to prolonged antibiotic use or treatment with multiple antibiotics. It has been demonstrated that there is a direct correlation between antibiotic use and dysbiosis of the gut microbiota. This condition, in turn, facilitates germination of and subsequent colonization by *C. difficile*, thereby establishing antibiotic exposure as the primary risk factor for CDI [9,10,11].

The gut microbiome plays a vital role in both the colonization of an individual by *C. difficile* and the onset of CDI. A healthy gut microbiome is characterized by the dominance of two phyla, Firmicutes and Bacteroidetes, and by high diversity and abundance of microorganisms. Additionally, it provides resistance to colonization by *C. difficile*. Specific families, such as *Bacteroidiaceae*, *Lachnospiraceae*, and *Ruminococcaceae,* protect against colonization of the intestine by *C. difficile*, while other families, such as *Enterococcaceae*, *Streptococcaceae*, and the phylum Proteobacteria, are associated with an increased risk of CDI [12]. Antibiotics reduce populations of beneficial bacteria, such as Bifidobacteria and Firmicutes, while increasing the abundance of Proteobacteria [13]. A person colonized with *C. difficile* does not necessarily develop the disease, as their intestinal microbiota protects them. Following antibiotic administration, the levels of bacteria that produce butyric acid (e.g., *Faecalibacterium prausnitzii*, *Eubacterium* spp., and *Roseburia* spp.) decline. The process described above has been shown to lead to an increase in inflammation, which, in turn, facilitates both spore germination and the onset of CDI [14].

This study presents an external validation cohort and is the first to examine the gut microbiomes of patients with CDI in comparison with those of healthy individuals in Greece. We attempted to examine whether the characteristic CDI microbiome disruption reported in international cohorts would also be observable in this population.

The primary objective of this study was to map and analyze the human gut microbiome in healthy individuals and patients with CDI. Furthermore, by comparing the microbiome cores of the two patient groups, we aimed to identify differences in the composition of the gut microbiota between CDI patients and healthy individuals and verify the concordance of our results with the current literature.

## 2. Materials and Methods

### 2.1. Study Design

We conducted a prospective study spanning March 2023 to October 2024 that included 48 residents of northern Greece who were over 18 years old. The participants were classified into two groups: 16 healthy individuals and 32 patients with CDI, namely, patients with diarrhea who tested positive for toxigenic *C. difficile*. All CDI patients were hospitalized at the time of diagnosis. All the participants provided written consent in accordance with the Declaration of Helsinki. Patient samples were collected after the onset of diarrhea.

### 2.2. Study Population

The volunteers who participated in the study had to meet specific criteria: they had to be over 18 years of age, they had to reside in northern Greece, they could not be participating in any other clinical studies, they could not be immunocompromised or have used corticosteroids in the last six months, and they could not have taken antibiotics, probiotics, or prebiotics in the six months prior to joining the study. We excluded individuals who had recently used antibiotics to reduce acute antibiotic-induced dysbiosis and better isolate microbiome alterations more specifically associated with CDI itself, rather than with recent antimicrobial pressure. Although this approach resulted in a more selective study population, it enhanced internal validity and improved the interpretability of microbiome–disease associations. The male-to-female ratio in the two groups was also set to be the same. These criteria were introduced to prevent other environmental factors, such as diet or medication, from influencing the results.

The medical history of both the healthy individuals and CDI patients included in this study was recorded in detail. Stool samples were collected from the patients, and laboratory confirmation of CDI was performed at the Microbiology Department of Aristotle University of Thessaloniki.

An episode of CDI was defined as a case with a positive toxigenic *C. difficile* test, along with diarrhea (≥3 loose stools in 24 h) or findings of pseudomembranous colitis, according to the definitions set out in the guidelines of the Society for Healthcare Epidemiology of America (SHEA) and the Infectious Diseases Society of America (IDSA) [15].

### 2.3. CDI Laboratory-Based Diagnosis

Samples were processed using a rapid detection kit for toxigenic *C. difficile*. Laboratory confirmation of CDI was performed using the enzyme immunoassay method (C Diff Quik-Chek Complete assay, TechLab, Blacksburg, VA, USA), which detected toxins A and B (produced by *C. difficile*) and glutamic dehydrogenase (GDH).

In addition, all samples that tested positive were cultured anaerobically on modified Brazier’s cycloserine–cefoxitin–egg yolk (CCEY) agar at 37 °C for 5 days, followed by recultivation of a colony on blood Columbia agar (CBA) for 48 h. Suspected cases of *C. difficile* colonies were confirmed via molecular amplification techniques for the genD gene encoding glutamate dehydrogenase and for the 16S rRNA gene [16,17].

Furthermore, all isolates were screened via PCR for the presence of toxin A (tcdA) and toxin B (tcdB) genes and the binary toxin (cdtA and cdtB). The oligonucleotide primers targeted the tcdA and tcdB genes within the pathogenicity locus operon (PaLoc) [18,19,20].

### 2.4. Gut Microbiome Analysis

The initial stool samples were then processed according to the protocol of the Human Microbiome Project (HMP) of the World Health Organization (WHO). Genetic isolation was performed using the DNeasy Power Soil Kit (QIAGEN, Valencia, CA, USA) [21]. The isolated DNA was subjected to PCR with the Ion 16S™ Metagenomics Kit (Life Technologies) to amplify the entire 16S region of the microbiome. For each sample, two sets of primers were used, V2-4-8 and V3-6-7-9, which allowed accurate detection and identification of a wide range of bacteria at the genus and species levels. The PCR product was purified with the AMPure XP reagent (Agencourt). Ion Torrent-compatible barcode adapters were ligated to the DNA using the Ion Xpress Plus Fragment Library Kit (Life Technologies, Carlsbad, CA, USA). Quantitative PCR was performed on the pooled barcoded libraries to assess quality and determine the dilution factor of the template for emulsion PCR. The pool of barcoded libraries was diluted appropriately and loaded into the Ion Chef System (Ion Torrent™, Thermo Fisher Scientific, Waltham, MA, USA) using materials from the Ion 510, Ion520, and Ion 530 Kit-Chef for automated template preparation and chip loading. The chips were Ion 520 (Ion Torrent™). The loaded chips were then loaded into the S5 sequencing system (Ion Torrent™) to complete the sequencing. This system uses sequencing technology with semiconductors. Unlike other NGS platforms, wherein the binding of nucleotides causes light production, this technology is based on changes in pH caused by the incorporation of a nucleotide into the sequencing chain. Primary data analysis was performed using Ion Reporter Software version 5.20.0.14.

### 2.5. Statistical Analysis

Statistical analysis was performed using R (version 3.5.2) [22]. The vegan (version 2.5.3), phyloseq (version 1.24.2), and ggplot2 (version 3.1.0) packages were used for data analysis and visualization [23,24,25]. The Wilcoxon rank-sum test was used to compare differences in the relative abundance of taxa and in alpha diversity (Shannon and Inverse Simpson indices) between patients and controls. Results with a *p*-value of less than 0.05 were considered significant. Also, a sensitivity power analysis of alpha diversity was performed using G*Power version 3.1.9.7 with a = 0.05 and power = 0.08. The MaAsLin2 tool was also used to identify significant differences in taxon abundance between groups [26]. The Benjamini–Hochberg method was used to adjust the *p*-values, and results with a *q*-value of less than 0.05 were considered significant. To investigate beta diversity, a Bray–Curtis dissimilarity matrix was calculated at the species level, and Principal Coordinate Analysis (PCoA) was performed to identify taxa contributing to overall variance. Permutational Multivariate Analysis of Variance (PERMANOVA) was performed with 1000 permutations [27]. Additionally, permutational analysis of multivariate dispersion (PERMDISP) was applied to further validate the PERMANOVA findings [28,29].

## 3. Results

### 3.1. Demographic Characteristics

During the study period, 48 stool samples were analyzed. Of these, 32 samples were obtained from patients with CDI, and 16 samples were obtained from healthy individuals. Among the patients, the male-to-female ratio was 0.6:1 (37.5% men and 62.5% women), while that in the healthy group was 0.5:1 (31.3% men and 68.7% women). The average age of men was 77 years (60–90 years) in the patient group and 80 years (73–86 years) in the healthy group. Similarly, the average age of women was 82 years (64–94 years) in the patient group and 76 years (58–82 years) in the healthy group. The corresponding demographic, anthropometric, and clinical data are provided in Table 1.

### 3.2. Abundance Analysis of Taxa

Eight phyla were detected across all samples. Among these, the phylum Deinococcus–Thermus was detected in only one patient (0.01%). The remaining seven were detected in both patients and controls. Firmicutes members were the most abundant in both the patients (42.21%) and controls (51.06%). In the patients, the second most abundant phylum was Proteobacteria (28.44%), followed by Bacteroidetes (23.13%). In contrast, in healthy controls, the second most abundant phylum was Bacteroidetes (24.23%), followed by Proteobacteria (18.03%). The non-parametric Wilcoxon rank-sum test did not detect any statistically significant differences in the abundances of phyla, as shown in Table 2.

**Figure 1:**
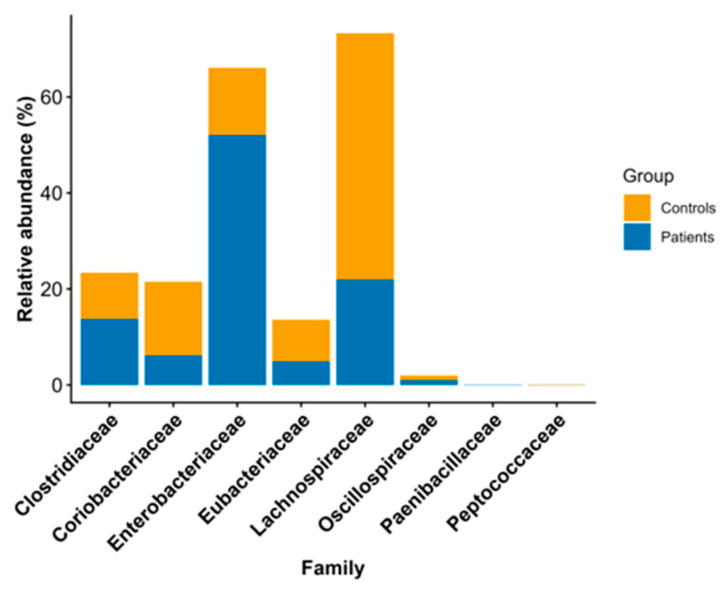
Stacked bar plot showing the relative abundances of statistically significant families among the controls and patients.

At the family level, in both groups, the first three families accounted for over 50% of the total microbiome composition. Specifically, the most abundant family among patients was *Enterobacteriaceae* (24.18%), followed by *Enterococcaceae* (21.40%) and *Bacteroidaceae* (13.45%). In contrast, *Ruminococcaceae* (23.98%) was the most abundant family in the controls, followed by *Lachnospiraceae* (20.58%) and *Bacteroidaceae* (13.66%). The non-parametric Wilcoxon rank-sum test identified statistically significant differences in eight families, as shown in Table 3 and depicted in Figure 1.

**Figure 2:**
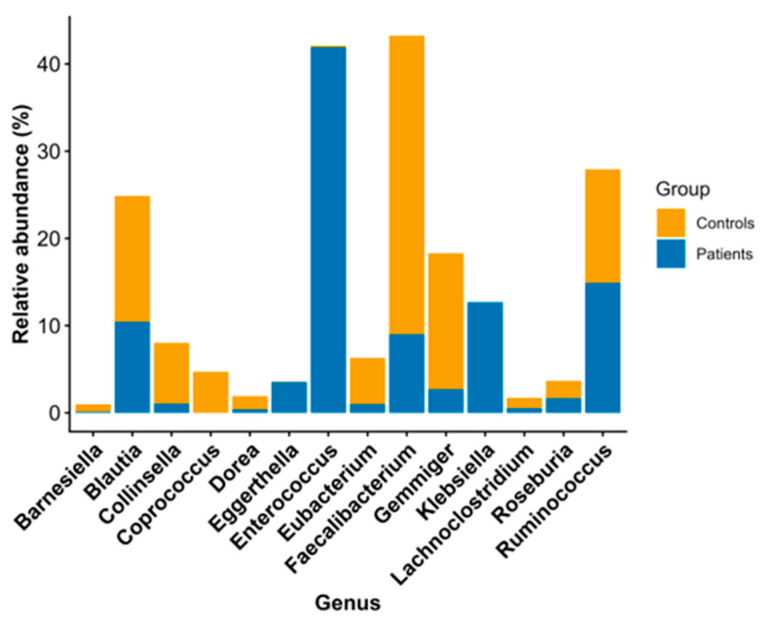
Stacked bar plot showing the relative abundances of statistically significant genera, grouped by family, between controls and patients.

At the genus level, *Enterococcus* was significantly more abundant in patients relative to the healthy controls (28.19%p vs. 0.17%c, *p*-value = 0.00002 < 0.05), while the abundances of *Blautia* (3.64%p vs. 14.03%c, *p*-value = 0.001 < 0.05), *Ruminococcus* (3.21%p vs. 8.49%c, *p*-value = 0.001 < 0.05), and *Faecalibacterium* (3.15%p vs. 17.76%c, *p*-value = 0.00001 < 0.05) were significantly lower in patients than in the healthy controls, as shown in Table 4 and depicted in Figure 2.

### 3.3. Analysis with MaAsLin2

The linearmodel applied using the MAAsLin2 tool identified additional taxa that showed significant differences between groups after multiple corrections (*q*-value < 0.05). The families *Hyphomicrobiaceae* (0.27%p vs. 8.55%c, effect size = −0.22, *q*-value = 0.00004) and *Ruminococcaceae* (3.13%p vs. 23.98%c, effect size = −0.36, *q*-value = 0.00004) and the species *Dorea longicatena* (0.0%p vs. 0.10%c, effect size = −0.02, *q*-value = 0.002) and *Coprococcus eutactus* (0.0%p vs. 1.65%c, effect size = −0.11, *q*-value = 0.03) were significantly less abundant in patients relative to the controls, as revealed by the negative effect size. In contrast, the family *Enterococcaceae* (21.4%p vs. 0.13%c, effect size = 0.31, *q*-value = 0.02) and the genera *Enterococcus* (28.19%p vs. 0.17%c, effect size = 0.35, *q*-value = 0.01) and *Serratia* (0.84%p vs. 0.0%c, effect size = 0.07, *q*-value = 0.03) were significantly more abundant in the patients relative to the controls, as revealed by the positive effect size. The data from the MaAsLin2 analysis are shown in Table 5.

Additionally, a multivariate linear regression model was constructed using MaAsLin2, incorporating age and gender as covariates. Following these adjustments, differential abundance analysis identified the taxa listed in Table 6. Age and gender were not significantly associated with the taxa identified.

### 3.4. Microbiome Diversity

**Figure 3:**
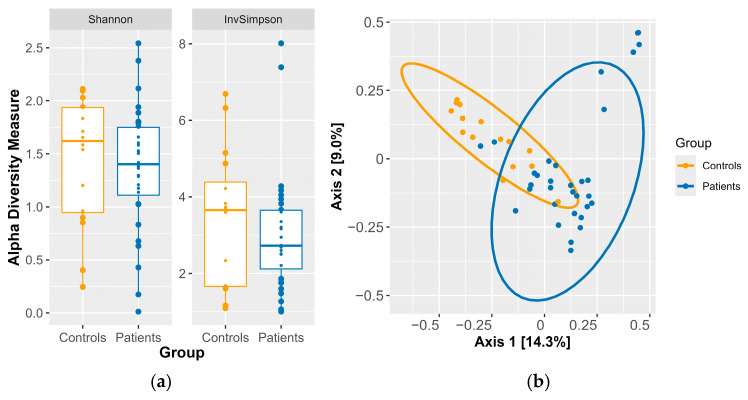
(**a**) Alpha diversity among the groups was explored using the Shannon and Inverse Simpson indices at the genus level. (**b**) PCoA was conducted to assess the overall structure of the gut microbiota at the species level in both patients and healthy controls.

The analysis of alpha diversity at the genus level did not reveal statistically significant differences for either the Shannon index (median = 1.4p vs. 1.6c, *p*-value = 0.7 > 0.05) or the Inverse Simpson index (median = 2.7p vs. 3.7c, *p*-value = 0.4 > 0.05) in patients relative to the controls, as depicted in Figure 3a.

**Figure 4:**
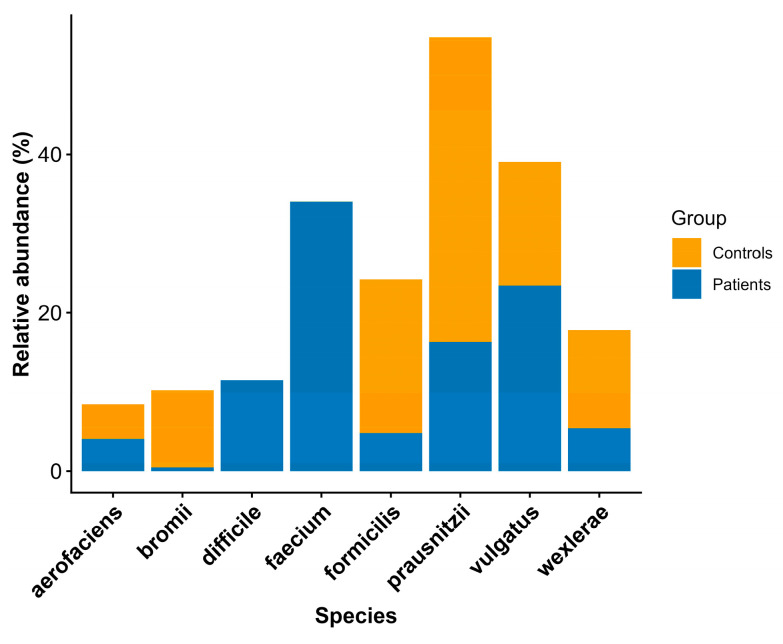
Stacked bar plot showing the relative abundances of statistically significant species between the controls and patients.

To assess beta diversity and identify differences in microbial community composition between patients and controls at the species level, we filtered out species that were detected with fewer than 30 reads in at least 5% of the samples. A PERMANOVA test with 1000 permutations confirmed that there were statistically significant differences in composition between the controls and patients (*p* = 0.0009 < 0.05). However, PERMDISP also revealed statistically significant differences in multivariate dispersion (*p* = 0.0009 < 0.05), indicating heterogeneity in within-group variability. As PERMANOVA is sensitive to differences in dispersion, the observed significance may reflect a combination of centroid separation and dispersion effects rather than purely compositional differences, as shown in Figure 3b. In Table 7 and Figure 4, we present the species that contributed to beta-diversity separation. However, according to the PERMANOVA and PERMDISP results, these species cannot be considered reliable indicators of group-specific microbial profiles. They are included due to their statistically significant differences in relative abundance and for exploratory analysis of abundance-associated microbial variation.

## 4. Discussion

CDI is a significant healthcare challenge worldwide that requires innovative solutions for effective management. Over the last few years, studies of the gut microbiome in patients with CDI have revealed a close relationship between specific microbial populations and the disease. Although no common core of intestinal microbiota characteristic of eubiosis has been identified, the abundance of the phyla Firmicutes and Bacteroidetes appears to be associated with a healthy intestinal microbiota and with the prevention of *C. difficile* colonization and CDI development [30]. In this study, we outline differences in the gut microbiome between patients with CDI and healthy individuals.

Eight phyla were found in the stool samples, seven of which were detected in both groups. Firmicutes was the dominant phylum in both groups studied, although its relative abundance was slightly lower in CDI patients. Firmicutes includes both beneficial bacteria families—*Lachnospiraceae* and *Ruminococcaceae*—and pathogens of the *Enterococcaceae* and *Streptococcaceae* families [31,32]. In CDI patients, there is a decline in the former and an increase in the latter, which explains why Firmicutes remains the dominant phylum in both groups. Moreover, the abundance of Proteobacteria among the patients in our study was greater than in the healthy group. The above changes are consistent with the literature, which indicates that individuals with a CDI exhibit an increase in Proteobacteria, which include many pathogenic bacteria, mainly from the family *Enterobacteriaceae* [10,33,34,35].

The families found in abundance in CDI patients in our study were *Enterobacteriaceae* and *Enterococcaceae*, while *Ruminococcaceae* and *Lachnospiraceae* showed reduced abundance in patients. In fact, for two of the above families—*Enterobacteriaceae* and *Lachnospiraceae*—we observed a statistically significant difference in relative abundance between the two groups. According to the literature, the family *Enterobacteriaceae* includes various pathogenic bacteria that are abundant under dysbiotic conditions, such as in CDI [32,34,36,37]. In contrast, the families *Ruminococcaceae* and *Lachnospiraceae* include bacteria that produce butyric acid, a short-chain fatty acid that inhibits the growth of *C. difficile* and prevents its colonization in the intestinal mucosa and subsequent development of CDI [32,38,39,40]. In a study by Zhou et al., the changes in the gut microbiome during the transition from a healthy state to *C. difficile* carriage and from carriage to the development of CDI were well described, and the role of butyric-acid-producing bacteria in CDI development was demonstrated [40]. According to their study, during the transition from non-carriage to colonization, there is an increase in the abundance of butyric-acid-producing bacteria among the intestinal microbiota, possibly as a response to colonization, in order to prevent CDI. In addition, the transition from a non-carrier state to CDI is accompanied by decreased bacterial diversity, a decreased abundance of butyric acid-producing bacteria, and an increased abundance of opportunistic pathogens of the *Enterobacteriaceae* family [40].

At the genus level, *Enterococcus* was found to be statistically significantly more abundant in the patients, whereas the relative abundance of *Faecalibacterium* was statistically significantly lower in patients than in healthy individuals. The genera *Blautia* and *Ruminococcus* also exhibited statistically significantly lower relative abundance among patients. Upon continuing the analysis at the species level, we found that the relative abundance of *Faecalibacterium prausnitzii* was statistically lower in patients than in healthy individuals.

It is known that the abundance of *Enterococcus* in the intestinal lumen reduces the number of beneficial bacteria, such as *Faecalibacterium*, *Bifidobacterium*, *Ruminococcaceae*, and *Lachnospiraceae*, and increases the likelihood of CDI [34]. Furthermore, it has been found that the presence of *Enterococcus* in abundance in the gut microbiota of individuals with CDI is associated with a poorer infection outcome due to the changes it causes in the intestinal environment [41]. Vakili et al. compared the gut microbiomes of individuals with CDI and healthy controls and found that the microbiomes of individuals with CDI showed an abundance of *Enterococcus*, *Lactobacillus*, *Escherichia coli*, *C. difficile*, and *Akkermansia muciniphila*. At the same time, the volumes of *Bacteroides*, *Bifidobacterium*, and *Faecalibacterium prausnitzii* were lower compared to the healthy control group [42]. Similarly, in a study by Gazzola et al., the gut microbiomes of individuals with CDI and rCDI were compared with the gut microbiomes of healthy individuals, and an increased ratio of *Enterococcus*, *Bacteroides*, and *Parabacteroides* was observed [43].

Martínez et al. examined the gut microbiomes of individuals with inflammatory bowel disease, CDI, and healthy controls. Once again, *Enterococcus* was found in high concentrations in the microbiomes of patients with CDI, alongside various other bacteria, including *Veillonella, Streptococcus*, *Escherichia–Shigella*, and *Enterobacteriaceae* [44]. In the same study, analysis at the species level revealed high concentrations of two *Enterococcus* species, *Enterococcus faecalis* and *Enterococcus faecium*, which are considered opportunistic pathogens, in the gut microbiomes of CDI patients [34]. However, the action of *Enterococcus faecalis* is controversial, as it was found to exhibit both probiotic and anti-*C. difficile* activity in another study [45].

The genera *Faecalibacterium*, *Ruminococcus,* and *Blautia* are beneficial bacteria of the Firmicutes phylum and are found at low levels in the gut microbiota of patients with CDI, as reported in many studies, including our study [31,33,46,47].

*Faecalibacterium prausnitzii* is a bacterium that produces butyric acid, and it is one of the most common bacteria in the gut microbiomes of healthy individuals; however, its abundance is lower in the microbiota of individuals with CDI. Many studies have investigated whether it plays a protective role against CDI and, if so, to what extent. A research article by Cassir et al. explored the relationship between CDI and *Faecalibacterium prausnitzii* and showed that *Faecalibacterium prausnitzii*’s protective role against CDI is independent of age and antibiotic use [48]. In another study by Bjorkqvist et al., an increase in the concentration of *Faecalibacterium prausnitzii* in the gut microbiota of patients with CDI was observed after fecal transplantation, and the concentration remained high for several months after transplantation [49].

The increase in the relative abundance of the family *Enterococcaceae* and the genus *Enterococcus* among patients was also confirmed by the MaAsLin2 model, which revealed higher relative abundance in patients than in healthy individuals for the microbes mentioned above. Additionally, we estimated the impact of gender and age on the abundance of certain taxa in the gut microbiome between the two study groups. We incorporated gender and age as covariates in a multivariate linear regression model using MaAsLin2. Although age and sex influence gut microbiota composition, they were not found to be significantly associated with the taxa identified in our study, suggesting that the observed differences in abundance are not driven by demographic variation between the groups.

It should also be noted that the investigation of alpha diversity at the genus level did not reveal statistically significant differences between the two groups. However, the Inverse Simpson index values for the healthy group were higher than those for the patient group. Thus, we can hypothesize that the intestinal microbiota of the healthy subjects in our study was probably more stable than that of the patients, in whom an imbalance tended toward a state of dysbiosis. However, this is only a theory and must be further investigated to allow us to draw reliable conclusions. The gut microbiomes of patients with CDI show reduced diversity compared with those of healthy individuals, as reported elsewhere [44,46,50]. Our study does not support this notion. However, our study is in alignment with a study conducted by Chen-See et al., who also found no differences in alpha diversity between patients with CDI and healthy individuals.

Regarding beta diversity, PERMANOVA identified significant differences in community structure between groups; however, the concurrent significance of PERMDISP indicates unequal multivariate dispersion, suggesting that the observed differences may reflect both shifts in community centroids and differences in within-group variability. The literature indicates that relative to healthy individuals, patients with CDI exhibit differences in beta diversity, which are due to differences in the composition of their intestinal microbiota [44,51]. Our results do not reflect this notion. The species *Enterococcus faecium* and *Faecalibacterium prausnitzii* were identified as contributors to group separation in beta diversity analyses; however, given the impact of multivariate dispersion on the overall beta-diversity results, these findings should be considered preliminary and require validation via larger studies. These species are nevertheless reported due to their statistically significant differences in relative abundance, providing exploratory insight into abundance-associated microbial variation.

## 5. Limitations

Our study was limited by the small number of samples included. For alpha diversity, the current sample provided sufficient power to detect large standardized mean differences (Cohen’s d ≥ 0.88), but smaller effects may have been missed. Given the multivariate nature of beta-diversity analyses and the multiple taxa-level comparisons in abundance analysis, the current sample size may have reduced statistical sensitivity, particularly for small to moderate effects. Therefore, the results of the beta diversity and abundance analyses should be considered exploratory and interpreted with caution. Nonetheless, the consistency observed across the diversity and abundance analyses provides preliminary evidence of the reported microbial patterns. Additionally, the results of our abundance analysis of taxa are consistent with the literature. Moreover, all our patients were elderly, and this fact may have had an impact on the composition of their gut microbiota. As mentioned in the Section 4, we tried to minimize this impact by adding age as a covariate in our multivariate statistical model. In addition, some healthy controls presented metabolic comorbidities, such as hypertension and diabetes mellitus, which have been associated with alterations in gut microbiota composition and could function as confounding factors. To address this issue, subgroup comparisons were performed within the control group, and no statistically significant differences were observed according to comorbidity status. Furthermore, the taxa that differed between the CDI patients and controls in our study—particularly the increased abundance of *Enterococcaceae* and *Enterococcus* and the reduction in the abundance of *Ruminococcaceae* and *Faecalibacterium*—are consistently reported as CDI-associated signatures in independent cohorts. Therefore, it is unlikely that the observed microbial shifts are solely attributable to underlying metabolic conditions. Nevertheless, residual confounding cannot be completely excluded, and larger studies with stratified analyses are required.

Another limitation was that it was not possible to obtain a stool sample before the onset of CDI. Consequently, we do not know whether a given patient was colonized with *C. difficile* before the infection. A further limitation of this study is the absence of data regarding the type of diet at the onset of CDI. In accordance with the findings of certain studies, an increase in fiber intake has been demonstrated to result in an increase in the abundance of beneficial microbes belonging to the genera *Bifidobacterium*, *Lactobacillus*, *Faecalibacterium*, *Roseburia*, and *Ruminococcus* within the gut microbiome [52,53]. In many cases, patients diagnosed with CDI reduce their fiber intake at the recommendation of their treating physician. Consequently, the decline in microbes belonging to the above genera observed in these patients’ microbiomes may not entirely be a consequence of CDI; it may also be influenced by reduced dietary-fiber intake. Finally, our study provides a detailed profile of taxonomic composition, but it lacks direct measurement of metabolic function or predictive functional profiling. Further studies using metabolomics or transcriptomics should be conducted to validate whether the observed structural changes translate into altered metabolic pathways and functional outputs.

## 6. Conclusions

In conclusion, our data show differences in microbiome patterns between healthy individuals and CDI patients. Specifically, CDI caused changes in the composition of the gut microbiome, with an increase in the number of specific bacterial taxa, including the family *Enterococcaceae* and the genus *Enterococcus*, and a reduction in the quantity of other bacterial taxa, such as the family *Ruminococcaceae* and the genus *Faecalibacterium*. These findings demonstrate that the characteristic CDI microbiome disruption reported in international cohorts is also observable in this population, supporting the generalizability of these microbial signatures rather than indicating a uniquely Greek microbiome profile. Further studies using larger sample sizes are necessary.

## Figures and Tables

**Table 1:** Demographic, anthropometric, and clinical data on the patients and healthy controls.

Sample Name	Sample Code	Group	Sex	Age (Years)	Nationality	Medical History
Patient 1	C1	P	F	94	Greek	FREE
Patient 2	C2	P	F	87	Greek	HYPO
Patient 3	C3	P	M	89	Greek	HF
Patient 4	C4	P	M	85	Greek	DM
Patient 5	C5	P	F	75	Greek	BP-DM-HYPO
Patient 6	C6	P	F	81	Greek	FREE
Patient 7	C7	P	F	88	Greek	BP-DM-HYPO
Patient 8	C8	P	F	86	Greek	BP
Patient 9	C10	P	F	68	Greek	DM
Patient 10	C11	P	F	77	Greek	BP-HYPO
Patient 11	C12	P	F	84	Greek	HF-DM
Patient 12	C13	P	M	90	Greek	BP-HF-HΥΠO
Patient 13	C15	P	M	80	Greek	BP
Patient 14	C16	P	F	88	Greek	BP-Afib
Patient 15	C17	P	F	87	Greek	BP
Patient 16	C19	P	M	75	Greek	BP
Patient 17	C20	P	F	82	Greek	DM
Patient 18	cd1	P	M	64	Greek	BP
Patient 19	cd2	P	F	67	Greek	BP
Patient 20	cd3	P	F	65	Greek	FREE
Patient 21	cd4	P	M	69	Greek	BP
Patient 22	cd5	P	F	82	Greek	HF
Patient 23	cd6	P	F	87	Greek	HF
Patient 24	cd7	P	F	83	Greek	HF
Patient 25	cd8	P	F	80	Greek	DM
Patient 26	cd9	P	M	60	Greek	FREE
Patient 27	cd10	P	M	78	Greek	BP
Patient 28	cd11	P	F	88	Greek	HF
Patient 29	cd12	P	M	76	Greek	BP
Patient 30	cd13	P	F	83	Greek	DM
Patient 31	cd14	P	M	79	Greek	BP
Patient 32	cd15	P	M	77	Greek	BP
Healthy control 1	N1	HC	F	82	Greek	DM-BP
Healthy control 2	N2	HC	M	82	Greek	HYPO
Healthy control 3	N3	HC	F	80	Greek	BP-HYPO
Healthy control 4	N4	HC	F	80	Greek	FREE
Healthy control 5	N5	HC	F	78	Greek	FREE
Healthy control 6	N6	HC	M	86	Greek	BP
Healthy control 7	N7	HC	F	67	Greek	BP
Healthy control 8	N8	HC	M	73	Greek	FREE
Healthy control 9	N9	HC	M	79	Greek	FREE
Healthy control 10	N10	HC	M	82	Greek	FREE
Healthy control 11	N11	HC	F	72	Greek	BP
Healthy control 12	N12	HC	F	82	Greek	BP
Healthy control 13	N13	HC	F	78	Greek	BP
Healthy control 14	N14	HC	F	78	Greek	FREE
Healthy control 15	N15	HC	F	80	Greek	DM-BP
Healthy control 16	N16	HC	F	58	Greek	BP

P—patient; HC—healthy control; M—male; F—female; BP—blood pressure; DM—diabetes mellitus; HF—heart failure; Afib—atrial fibrillation; Hypo—hypothyroidism; and FREE—free medical history.

**Table 2:** Abundance (counts) and relative abundance (%) of phyla in patients and controls.

Phylum	# Patients	% Patients	# Controls	% Controls	*p*-Value
Firmicutes	1,473,494	42.21	500,000	51.06	0.17
Proteobacteria	992,886	28.44	176,534	18.03	0.32
Bacteroidetes	807,415	23.13	237,337	24.23	0.69
Actinobacteria	164,312	4.71	65,204	6.66	0.23
Verrucomicrobia	39,193	1.12	78	0.01	0.54
Fusobacteria	13,157	0.38	25	<0.01	0.32
Deinococcus–Thermus	282	0.01	Not detected	Not detected	-
Synergistetes	193	0.01	142	0.01	0.38

# as counts.

**Table 3:** Abundance (counts) and relative abundance (%) of families with statistically significant differences between patients and controls.

Phylum	Family	# Patients	% Patients	# Controls	% Controls	*p*-Value
Firmicutes	*Lachnospiraceae*	229,598	6.58	201,566	20.58	0.001
	*Clostridiaceae*	63,622	1.82	21,779	2.22	0.04
	*Eubacteriaceae*	10,577	0.3	20,709	2.11	0.0002
	*Oscillospiraceae*	9696	0.3	1543	0.16	0.02
	*Paenibacillaceae*	1152	0.03	11	0.001	0.01
	*Peptococcaceae*	49	0.001	320	0.03	0.02
Proteobacteria	*Enterobacteriaceae*	844,228	24.18	90,659	9.26	0.007
Actinobacteria	*Coriobacteriaceae*	40,185	1.15	53,910	5.5	0.008

# as counts.

**Table 4:** Abundance (counts) and relative abundance (%) of genera with statistically significant differences between patients and controls.

Family	Genus	# Patients	% Patients	# Controls	% Controls	*p*-Value
*Coriobacteriaceae*	*Eggerthella*	21,058	0.82	315	0.13	0.01
	*Collinsella*	14,567	0.56	40,935	5.56	0.00002
*Porphyromonadaceae*	*Barnesiella*	415	0.02	603	0.08	0.01
*Enterococcaceae*	*Enterococcus*	727,906	28.19	1204	0.17	0.00002
*Eubacteriaceae*	*Eubacterium*	4553	0.18	15,751	2.17	0.00001
*Lachnospiraceae*	*Blautia*	93,944	3.64	101,693	14.03	0.001
	*Roseburia*	5794	0.22	6799	0.94	0.0002
	*Dorea*	2304	0.09	7051	0.97	0.00001
	*Lachnoclostridium*	1167	0.05	3233	0.45	0.03
	*Coprococcus*	74	0.003	10,051	1.39	0.00002
	*Ruminococcus*	82,897	3.21	61,506	8.49	0.001
*Ruminococcaceae*	*Faecalibacterium*	81,252	3.15	128,683	17.76	0.00001
*Enterobacteriaceae*	*Klebsiella*	67,866	2.63	14	0.002	0.0002
*Hyphomicrobiaceae*	*Gemmiger*	9382	0.36	83,750	11.56	0.0000003

# as counts.

**Table 5:** MaAsLin2 analysis identified specific taxa that were significantly less or more abundant in patients than in the controls, with a *q*-value < 0.05. The taxa highlighted in bold were also identified as statistically significant based on the Kruskal–Wallis test during the abundance analysis.

**Family**	**Effect Size**	***p*-Value**	***q*-Value**	**% Patients**	**% Controls**
*Hyphomicrobiaceae*	−0.22	0.000003	0.00004	0.27	8.55
*Ruminococcaceae*	−0.36	0.000002	0.00004	3.13	23.98
***Eubacteriaceae***	−0.10	0.0001	0.001	0.30	2.11
*Enterococcaceae*	0.31	0.004	0.02	21.40	0.13
Genus	Effect Size	*p*-value	*q*-value	% Patients	% Controls
***Gemmiger***	−0.25	0.000001	0.00005	0.36	11.56
***Faecalibacterium***	−0.31	0.00001	0.0001	3.15	17.76
***Eubacterium***	−0.12	0.00003	0.0004	0.18	2.17
***Coprococcus***	−0.10	0.001	0.01	0.003	1.39
***Dorea***	−0.06	0.002	0.01	0.09	0.97
***Collinsella***	−0.10	0.004	0.02	0.56	5.56
***Roseburia***	−0.06	0.01	0.02	0.22	Controls
*Enterococcus*	0.35	0.001	0.01	28.19	0.17
*Serratia*	0.07	0.01	0.03	0.84	0.0
Species	Effect Size	*p*-value	*q*-value	% Patients	Controls
***Gemmiger* *formicilis***	−0.28	0.000001	0.0001	0.58	14.81
***Faecalibacterium* *prausnitzii***	−0.33	0.00001	0.0003	4.43	19.82
***Ruminococcus* *bromii***	−0.16	0.0001	0.002	0.09	4.07
*Dorea longicatena*	−0.02	0.0001	0.002	0.0	0.10
***Ruminococcus* *gauvreauii***	−0.05	0.0001	0.002	0.003	0.05
***Ruminococcus* *faecis***	−0.03	0.001	0.01	0.04	0.19
***Roseburia* *faecis***	−0.07	0.001	0.02	0.02	0.95
*Coprococcus eutactus*	−0.11	0.003	0.03	0.0	1.65

**Table 6:** Taxa significantly associated with disease status were identified using MaAsLin2, with adjustments for age and gender. Although age and gender were included as covariates, neither was significantly associated with any of the reported taxa.

**Family**	**Metadata**	**Effect Size**	***p*-Value**	***q*-Value**
*Ruminococcaceae*	Patient	−0.36	0.000003	0.0002
*Hyphomicrobiaceae*	Patient	−0.22	0.000006	0.0002
*Eubacteriaceae*	Patient	−0.09	0.0002	0.004
Genus	Metadata	Effect Size	*p*-value	*q*-value
*Gemmiger*	Patient	−0.25	0.000002	0.0003
*Faecalibacterium*	Patient	−0.31	0.000009	0.0005
*Eubacterium*	Patient	−0.11	0.0001	0.003
Species	Metadata	Effect Size	*p*-value	*q*-value
*Gemmiger formicilis*	Patient	−0.28	0.000001	0.0003
*Faecalibacterium prausnitzii*	Patient	−0.33	0.00001	0.002
*Ruminococcus bromii*	Patient	−0.17	0.0001	0.01
*Ruminococcus gauvreauii*	Patient	−0.05	0.0001	0.01
*Dorea longicatena*	Patient	−0.02	0.0003	0.02

**Table 7:** Speciesthat contribute to differences between groups, along with the statistical differences in abundance.

Species	# Patients	% Patients	# Controls	% Controls	*p*-Value
*Enterococcus faecium*	243,390	15.14	34	0.01	0.002
*Bacteroides vulgatus*	104,596	6.51	77,402	13.68	0.01
*Faecalibacterium prausnitzii*	71,260	4.43	112,106	19.82	0.000004
*Blautia wexlerae*	20,046	1.25	35,207	6.22	0.0005
*Collinsella aerofaciens*	13,227	0.82	25,934	4.59	0.0001
*Gemmiger formicilis*	9382	0.58	83,750	14.81	0.0000004
*Ruminococcus bromii*	1405	0.09	23,028	4.07	0.0000007

# as counts.

## Data Availability

The data presented in this study are available on request from the corresponding author.
